# Electrochemical Atomic Force Microscopy Study on the Dynamic Evolution of Lithium Deposition

**DOI:** 10.3390/ma16062278

**Published:** 2023-03-12

**Authors:** Xixiu Shi, Jingru Yang, Wenyang Wang, Zhaoping Liu, Cai Shen

**Affiliations:** 1 Ningbo Institute of Materials Technology & Engineering Chinese Academy of Sciences, 1219 Zhongguan Road, Zhenhai District, Ningbo 315201, China; 2 College of Materials Science and Opto-Electronic Technology, University of Chinese Academy of Sciences, Beijing 100049, China; 3 Department of Chemical and Environmental Engineering, University of Nottingham Ningbo China, 199 Taikang East Road, Ningbo 315100, China; 4 China Beacons Institute, University of Nottingham Ningbo China, 211 Xingguang Road, Ningbo 315100, China

**Keywords:** lithium metal, lithium dendrite, in situ EC-AFM, lithium deposition schematic

## Abstract

Lithium metal is one of the most promising anode materials for lithium-ion batteries; however, lithium dendrite growth hinders its large-scale development. So far, the dendrite formation mechanism is unclear. Herein, the dynamic evolution of lithium deposition in etheryl-based and ethylene carbonate (EC)-based electrolytes was obtained by combining an in situ electrochemical atomic force microscope (EC-AFM) with an electrochemical workstation. Three growth modes of lithium particles are proposed: preferential, merged, and independent growth. In addition, a lithium deposition schematic is proposed to clearly describe the morphological changes in lithium deposition. This schematic shows the process of lithium deposition, thus providing a theoretical basis for solving the problem of lithium dendrite growth.

## 1. Introduction

Lithium metal is considered the next-generation anode material for lithium-ion batteries due to its ultrahigh theoretical specific capacity (3860 mAh/g) and low electrochemical potential (3.04 V vs. a standard hydrogen electrode) [1,2]. However, dendrite growth severely hinders its commercialization. Several methods have been proposed to solve this problem, such as electrolyte engineering [3,4,5,6,7,8,9,10,11,12,13,14], planting lithophilic sites [1,15,16,17,18], 3D current collectors [19,20], artificial solid electrolyte interfaces (SEIs) [21,22,23,24], etc. All these methods inhibit the growth of lithium dendrites and thus significantly extend the cycle life of lithium-metal batteries [25,26,27,28]. Nevertheless, these methods cannot eliminate the lithium dendrite problem completely, and there are still some potential security risks in the long-term operation of lithium metal batteries.

Understanding the lithium dendrite formation mechanism is necessary to resolve this problem completely. The origin of lithium dendrites is inhomogeneous lithium deposition [29]. Accordingly, the key to understanding lithium dendrite growth is to clarify the lithium deposition process. Many efforts have been made to investigate this mechanism [30,31], especially in solid electrolytes [32,33]. However, many powerful and advanced characterization instruments cannot be directly used to observe in situ the lithium deposition in liquid electrolytes, due to the existence of liquid electrolytes. Many previous studies were carried out by depositing a certain amount of lithium on a substrate, drying it, and transferring it to the characterization instrument [34,35,36,37]. Based on this research method, lithium deposition is roughly divided into three regions according to the current density. Lithium deposition presents a uniform, dispersed, and nearly spherical deposition morphology under an SEI film at an extremely low current density [38]. With the increase of current density, it is found that the Li^+^ transport ability of SEI is difficult to keep up with the increase of current density, indicating a mixed diffusion−reaction control process. And it leads to the deposition morphology between spherical and filamentous. The SEI is broken when the current density continues to increase, creating a filamentous lithium dendrite morphology [39]. Finally, the lithium deposition morphology is rhombic dodecahedral at an ultrahigh current density since the SEI is destroyed and rhombic dodecahedra are characteristic of {110}-faceted body-centered cubic (BCC) crystals, and (110) are the closest packed planes in BCC metals including Li [29,36]. It is noteworthy that an optical microscope can be used to observe lithium dendrite growth in liquid electrolytes in situ and in real-time [40,41], but its accuracy can only reach the micron level at present, which cannot meet the needs of lithium deposition characterization at a nanoscale resolution. Consequently, the dynamic process of lithium deposition in liquid electrolytes is unclear due to the lack of direct evidence.

As an advanced and powerful characterization tool, atomic force microscopy (AFM) provides a three-dimensional morphology in a liquid environment [42,43,44,45]. Previously, in situ electrochemical atomic force microscopy (EC-AFM) has been utilized to observe SEIs’ morphological and surface property evolutions in operando conditions at a nanoscale resolution without damaging the fragile samples [31,46,47,48,49]. The lithium deposition behavior on graphite was obtained using in situ EC-AFM and used as a reference for studying lithium deposition [50].

Accordingly, the in situ and real-time characterization of lithium deposition in liquid electrolytes at a nanoscale resolution using in situ electrochemical atomic force microscopy may provide us with a new and more detailed perspective to help us understand the morphological evolution process of lithium deposition.

Herein, with the help of in situ EC-AFM, we successfully obtained dynamic evolution information on the lithium deposition process in situ and in real-time, which makes up for the lack of previous work. Furthermore, according to the dynamic evolution information on lithium deposition, the morphological evolution law for the lithium deposition process is summarized, and a lithium deposition schematic is proposed from the two processes of lithium metal nucleation and growth. In the nucleation process, the overpotential is the main factor affecting the morphology. In the growth stage, the mechanism of the longitudinal growth of individual particles and three growth modes are proposed by tracking the growth process of the particles. This work can help us to understand the lithium deposition process better, thus providing a theoretical basis for understanding and solving the lithium dendrite problem in the future.

## 2. Experimental Section

### 2.1. Preparation of Electrolytes

The electrolytes used in this study were 1M lithium bis(trifluoromethanesulphonyl)imide (LiTFSI) in 1,3-dioxolane (DOL):1,2-dimethoxyethane (DME) (1:1 by volume), 1 M LiTFSI in DOL:DME (1:1 by volume) with 2% lithium nitrate (LiNO_3_), and 1 M lithium hexafluorophosphate (LiPF_6_) in ethylene carbonate (EC):dimethyl carbonate (DMC) (1:1 by volume). The components of the electrolytes and corresponding marks are shown in Table 1; both were purchased from the DodoChem, Suzhou, China.

### 2.2. In Situ Electrochemical Atomic Force Microscopy

The in situ EC-AFM (Bruker Icon) was placed inside a glovebox (MBRAUN; H_2_O ≤ 0.1 ppm; O_2_ ≤ 0.1 ppm) and operated with ScanAsyst in fluid mode. Chronopotentiometry (CP) was performed at room temperature (20 °C) using an electrochemical workstation to investigate the constant current deposition. T2 copper (purchased from Taobao) was used as a working electrode (WE) for lithium deposition. Abrasive 180-mesh, 240-mesh, 600-mesh, 800-mesh, 1000-mesh, 1200-mesh, 1500-mesh, 2000-mesh, 2500-mesh, 3000-mesh, 5000-mesh, and 7000-mesh papers were used to polish the copper plate from a rough surface to a smooth one, and then it was cleaned ultrasonically in water and, finally, blow-dried with nitrogen. A lithium strip was employed as a counter and reference electrode (CE and RE) to construct a three-electrode system in an electrochemical cell. Constant-current deposition was performed, and EC-AFM was operated to obtain in situ dynamic evolution images. Furthermore, ScanAsyst in fluid mode, with the SCANNASYST-FLUID+ probe, was employed to study the topographies. Kelvin probe force microscopy (KPFM) was utilized to obtain the relative surface potentials of the dried samples at an imaging scan rate of 1 Hz per line and a resolution of 256 × 256 pixels.

### 2.3. Battery Preparation and Electrochemical Test

Cu foil was assembled using a lithium sheet to form CR2032 coin cells. The electrochemical workstation (CHI 760E, Chenhua, Shanghai, China) was used to monitor the chronopotentiometric (CP) and chronopotentiometry (CA) of the Cu||Li cells and the Tafel curve of the Li||Li cells. The current density for the CP measurement was 0.5 mA/cm^2^. The CA measurement voltage was −0.05 V. The Tafel measurements were carried out in the voltage range of −0.3 V to 0.3 V at a 0.001 V/s scan rate.

## 3. Results and Discussion

### 3.1. Nucleation Process

**Figure 1:**
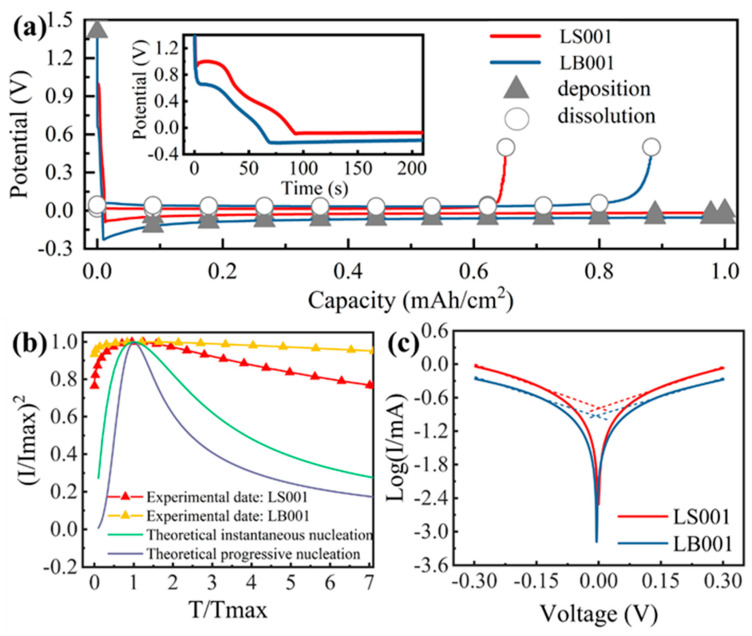
(**a**) Electrochemical deposition and dissolution curves for LB001 and LS001 at 0.5 mA/cm^2^; the insert shows the lithium nucleation. (**b**) Dimensionless graphs of current transients in Li metal deposition on Cu at −0.05 V. (**c**) Tafel curves for LB001 and LS001.

Figure 1a shows the electrochemical curve of the lithium deposition on the Cu substrate in the LS001 and LB001 electrolytes. The lithium deposition in the two electrolytes shows the same deposition process at a current density of 0.5 mA/cm^2^, detailed as follows: The first step is the electric double layer (EDL) charging process, whereby the electrode potential decreases continuously, accompanied by the formation of the initial SEI. The second step is lithium nucleation. The current transient curves of LS001 and LB001 are similar to the theoretical curve of transient nucleation [35,51] (Figure 1b), wherein the electrode potential decreases to the nucleation potential, instantly forming lithium nuclei on the Cu surface. Finally, the potential rises and enters the growth process.

Although the lithium deposition process in both electrolytes is the same, the deposition parameters are significantly different. The nucleation potential of the LB001 electrolyte is −0.2279 V, while that of the LS001 electrolyte is only –0.0898 V. The LB001 electrolyte has a higher nucleation overpotential than the LS001 electrolyte. Before the electrode potential lowers to the nucleation potential, LS001 consumes electrolytes with a capacity of 0.0129 mAh/cm^2^, while LB001 consumes fewer electrolytes (at 0.0106 mAh/cm^2^), which is related to the difficulty in the polarization of the two electrolytes. The Tafel curves show that the current exchange density (i_0_) in LB001 is smaller than that in LS001, suggesting that LB001 is more prone to polarization, so the required potential is reached more quickly.

The effects of nucleation overpotential and exchange current density on lithium nucleation morphology were studied.

**Figure 2:**
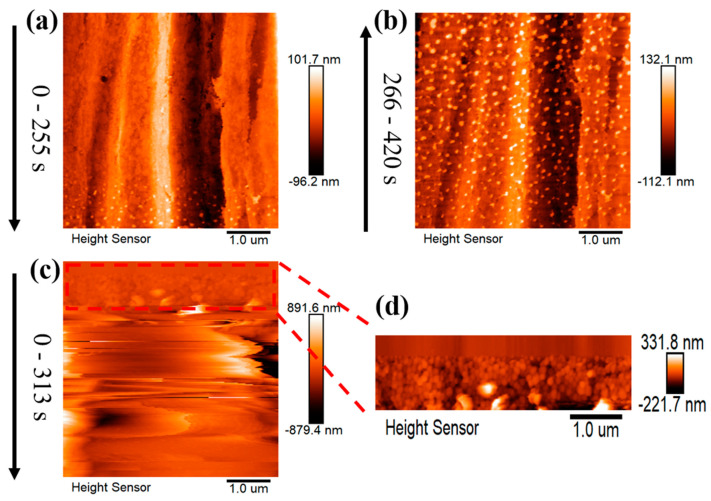
(**a**,**b**) In situ EC-AFM images of lithium nucleation in LS001 at different deposition times. (**c**) In situ EC-AFM images of lithium nucleation in LB001. (**d**) The details in the red box of Figure 2c. The deposition time is on the left side of the images, and the current density is 0.5 mA/cm^2^.

In situ EC-AFM explores the dynamic evolution of lithium nucleation, as shown in Figure 2. The most significant difference between the two electrolytes is in the density and distribution of lithium nuclei. LB001, with a higher nucleation overpotential, exhibits a finer and denser nucleation morphology (Figure 2d). In comparison, LS001, with a lower nucleation overpotential, shows fewer and more dispersed lithium nucleation (Figure 2a), suggesting that nucleation overpotential can be used to describe lithium nucleation in different electrolytes, and this effect is consistent with that in the same electrolyte: a large overpotential leads to fine and dense lithium nucleation, and a small overpotential leads to dispersed nucleation.

A higher exchange current density indicates more difficult polarization, suggesting that it may take longer to reach the required nucleation potential in the EDL charging process. As we can see in Figure 2a, in the LS001 electrolyte with the higher exchange current density, it takes longer for obvious lithium nuclei to appear, while in the LB001 electrolyte, obvious lithium nuclei appear faster (Figure 2d). In other words, before lithium nucleation, the surface of the Cu is exposed to the LS001 electrolyte for a longer time.

**Figure 3:**
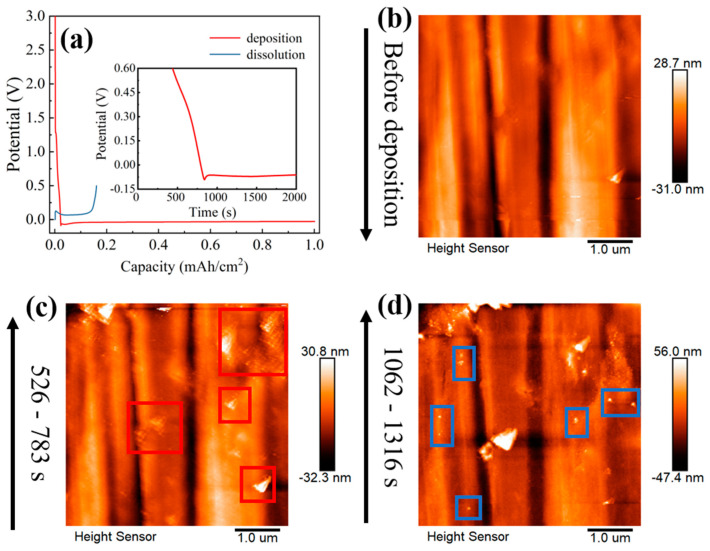
(**a**) Electrochemical deposition and dissolution curve in LB001 at 0.1 mA/cm^2^; the insert is the lithium nucleation. (**b**) EC-AFM image of Cu surface before deposition. (**c**,**d**) In situ EC-AFM images of lithium deposition at different deposition times. The deposition time is on the left side of the images, and the current density is 0.1 mA/cm^2^.

In order to clearly observe the effect of this long-term exposure of the surface of the Cu, we significantly extended the EDL charging time by reducing the deposition current density of LB001 to 0.1 mA/cm^2^, and the result is shown in Figure 3. At a low current density of 0.1 mA/cm^2^, the EDL charging process takes about 840 s to reach the required nucleation potential (Figure 3a), indicating the formation of an initial SEI with a 0.0233 mAh/cm^2^ capacity on the Cu substrate interface, and the initial SEI is visible on the Cu surface (see the red box in Figure 3c). The long-term exposure of the Cu surface consumes more electrolytes and forms a thicker initial SEI, which is a reason for the significant decrease in the Coulomb efficiency (Figure 1a and Figure 3a). Furthermore, the lower current density also reduces the nucleation potential. In LB001, the nucleation potential at 0.1 mA/cm^2^ (−0.0910 V) is similar to that in LS001 at 0.5 mA/cm^2^ (−0.0898 V). Thus, the nucleation morphology in LB001 at 0.1 mA/cm^2^ is similar to that in LS001 at 0.5 mA/cm^2^. Interestingly, both exhibit rare, dispersed nucleation and slow growth (see Figure 1a and the blue box in Figure 3d).

A low exchange current density and a high nucleation overpotential for lithium nucleation are what we expect, representing the nucleation potential being reached more quickly and a smaller, denser nucleation morphology.

### 3.2. Lithium Nuclei Growth Process

The next process after lithium nucleation is the growth of lithium nuclei. The dynamic evolution of the growth process is observed using in situ EC-AFM. The growth rate of lithium nuclei in LB001 is too fast to capture images (Figure 2c). Therefore, it is the growth of lithium nuclei in LS001 that is mainly observed here.

**Figure 4:**
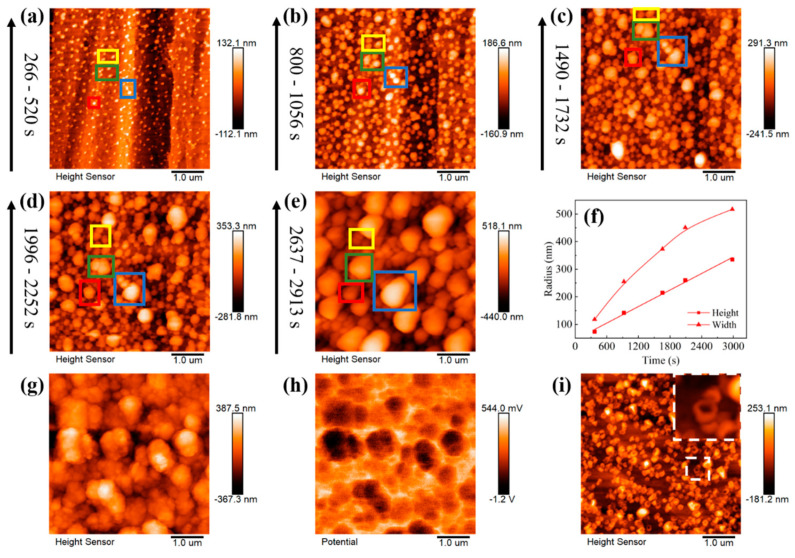
(**a**–**e**) In situ EC-AFM images of lithium particle growth in LS001 at 0.5 mA/cm^2^. (**f**) Horizontal and vertical growth curves for selected particle. The morphology (**g**) and the relative surface potential image (**h**) after deposition and drying. (**i**) Dissolution morphology after deposition in LS001.

**Figure 5:**
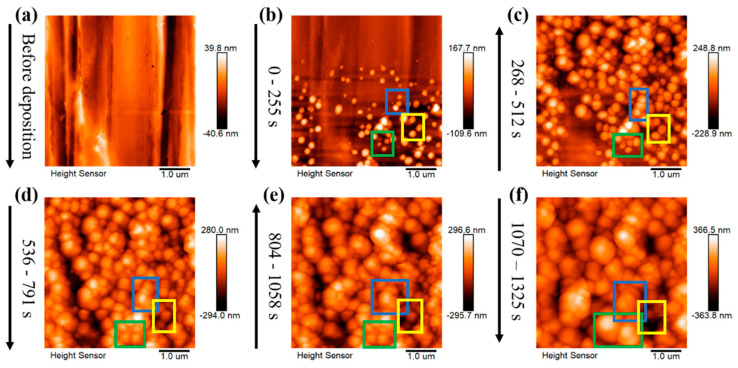
(**a**–**f**) In situ EC-AFM images of particle growth at different time in LS009 at 0.5 mA/cm^2^, and the deposition time is on the left side of the images. Different color boxes represent different growth modes: blue box: preferential growth; green box: merged growth; yellow box: independent growth.

Figure 4 shows the dynamic evolution of lithium nuclei growth in LS001. There is a certain gap between the lithium nuclei, without any obvious interaction between the lithium nuclei growths. Then, the lithium nuclei gradually grow into particles with the increase in the deposition capacity, and the gaps between the lithium particles gradually narrow; thus, the interactions between the lithium particles gradually increase until the particles are in direct contact. Since different particles show different growth modes after connecting, three modes are summarized herein. First, when particles are connected, the particles that originally grew slowly are further inhibited, slowing down the growth or even stopping it, whereas the lithium particles that grew faster grow faster than before. However, the boundaries between the particles are still clearly visible, and the particles are not fused; therefore, several smaller particles finally surround a large particle. This mode is marked as preferential growth (see the blue box in Figure 4a–e). The second growth mode is when several particles are connected, whereby they still grow together at a similar rate instead of in the preferential growth mode, squeezing each other until they merge into a large particle (see the green box in Figure 4a–e). This mode is marked as merged growth. Finally, some particles do not grow preferentially or merge to form a large particle but exist independently. However, it is difficult for particles in this mode to grow very fast, and they may be covered by or merged with other large particles (see the yellow box in Figure 4a–e). With the increase in the deposition capacity, the large particles are adjacent to other large particles and repeat the above process. Finally, a dense sedimentary layer is formed. These three modes are also observed in LS009, as shown in Figure 5, and the modes represented in the colored boxes in Figure 5 are consistent with Figure 4a–e, indicating that these modes are not accidental results but repeatable lithium nucleus growth laws. Interestingly, the particles we were tracking that were growing in independent growth mode are gradually covered by other particles (see the yellow box in Figure 5b–e), and almost disappear in Figure 5f, which is exactly what we predicted above.

To compare the growth rates of individual particles in the horizontal and vertical directions under a small deposition capacity, the height and width of the individual particles selected in the red box in Figure 4a–e were measured at different deposition times (Figure 4f). In the early stage, the growth rate in the horizontal direction is similar to that in the vertical direction. However, the growth rate decreases in the horizontal direction when the gap between lithium nuclei decreases. In other words, particles are inclined to grow taller rather than fatter. The space limitation in the horizontal direction inhibits growth in the horizontal direction, while there is no space limitation in the vertical direction. However, according to the relative surface potentials of the lithium particles obtained via KPFM, a new reason is proposed. The relative surface potentials at the tops and sides of the lithium particles are greatly different (Figure 4h). The relative surface potential of the SEI is low at the top of most particles, while it is high at the side, indicating that the work function of the SEI at the top of particles is higher than that at the side. The SEI at the top of particles with higher work functions has a higher electron binding energy; thus, the electrons cannot pass through the SEI, suggesting that the top cannot decompose the electrolyte, facilitating Li^+^ transport. In contrast, the SEI at the side of a particle decomposes the electrolyte easily and forms the SEI, thus inhibiting growth at the side. The slow rate of side growth aggravates the formation of the SEI, a positive feedback loop, decreasing the growth at the side until it stops. The difference in the SEI between the top and side creates the difference in the lithium nuclei growth in the different directions and affects the lithium-particle dissolution. Since the SEI at the top is more conducive to Li-ion passage, it is easier for lithium metal to dissolve from the top during the dissolution stage, forming a doughnut-shaped dissolution morphology (Figure 4i), which is consistent with the work previously reported [52].

**Figure 6:**
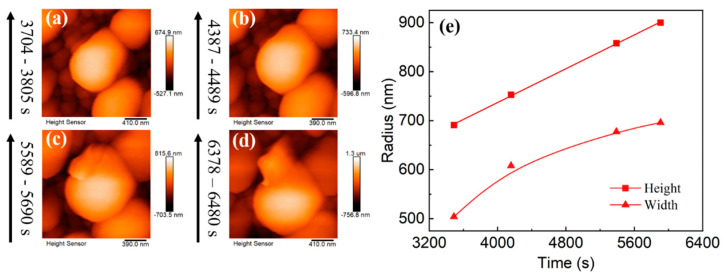
(**a**–**d**) In situ EC-AFM images of selected particles’ growth in LS001 at 0.5 mA/cm^2^. (**e**) Horizontal and vertical growth curves for selected particles. The deposition time is on the left side of the images.

**Figure 7:**
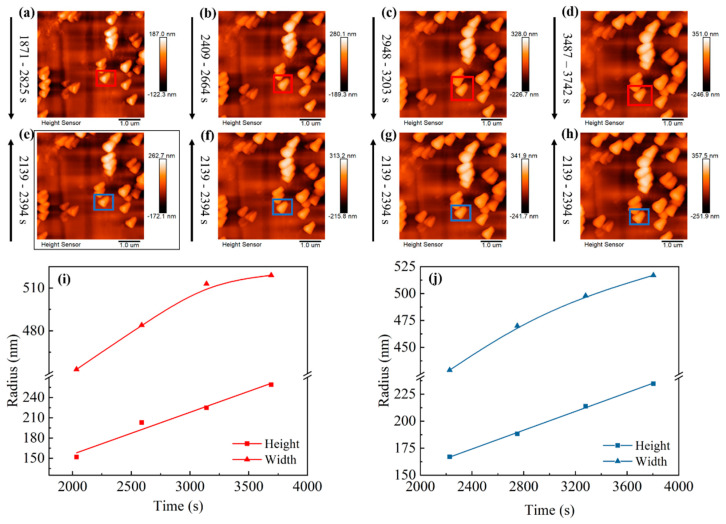
In situ EC-AFM images of lithium deposition in LB001 at 0.1 mA/cm^2^: (**a**–**d**) capture direction, down; (**e**–**h**) capture direction, up. Horizontal and vertical growth curves for selected particles in different capture directions: (**i**) capture direction, down; (**j**) capture direction, up. The deposition times are written below the images.

To verify that the particles still tend to grow higher under a larger deposition capacity, the particle marked in the blue box in Figure 4a–e was selected, and the particle’s height and width were measured (Figure 6). As expected, even under a high deposition capacity, the growth of the particles is still consistent with the rule mentioned above. The growth rate in the horizontal direction gradually decreases while that in the vertical direction is unchanged. Furthermore, an obvious prelude to lithium dendrite growth is observed on selected particles (Figure 5a–d). Similarly, the same conclusion was reached by measuring the height and width of particle growth in LB001 at 0.1 mA/cm^2^ (Figure 7).

**Figure 8:**
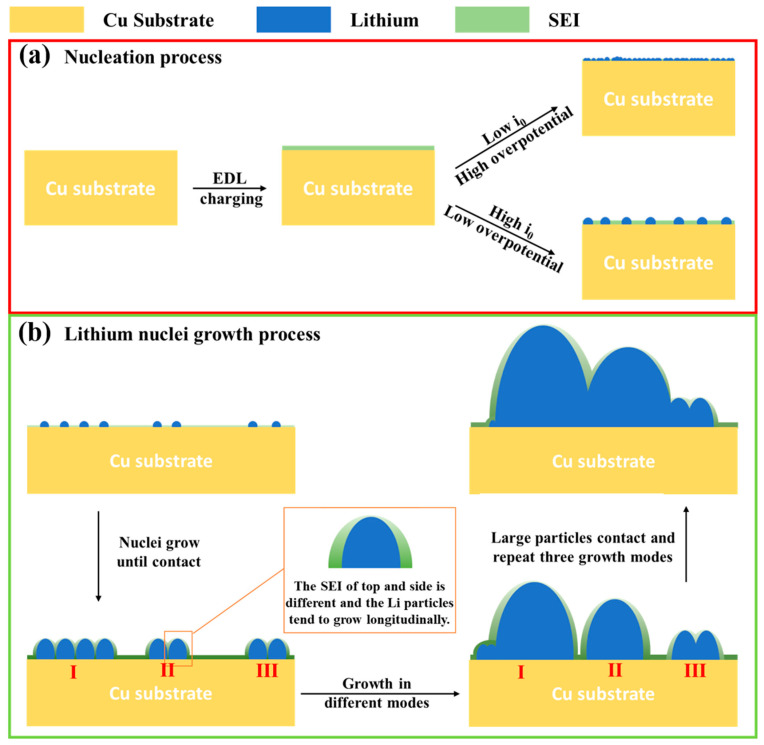
Schematic diagrams of lithium nucleation and growth.

In conclusion, dynamic evolution images of lithium nucleation and growth in LS001, LB001, and LS009 were obtained using in situ EC-AFM. A schematic of lithium nucleation and growth was proposed and shown in Figure 8. Firstly, the effect of exchange current density and nucleation overpotential on nucleation morphology was discussed for different electrolytes. This schematic shows that electrolytes with a low exchange current density and high nucleation overpotential cause dense and fine nucleation. In contrast, electrolytes with a high exchange current density and low nucleation overpotential cause dispersed nucleation (Figure 8a). Secondly, three growth modes in the growth process were proposed, as shown in Figure 8b. Mode I was marked as preferential growth, indicating that one of the particles will grow at a faster rate. In contrast, the growth of other particles is inhibited. Mode II was marked as merged growth, such that several particles squeeze each other to form a large particle. Finally, some particles do not grow preferentially or merge to form a large particle and exist independently. The height and width of an individual particle were measured at different deposition times, indicating that the growth rate in the vertical direction is almost unchanged; however, the growth rate in the horizontal direction decreases, creating a long strip deposition morphology. This mechanism is related to the difference between the top and sides of the SEI, inferred from the different surface potentials between the top and sides of the SEI.

## 4. Conclusions

Using the combination of in situ EC-AFM with an electrochemical workstation, lithium deposition’s dynamic morphology evolution was obtained from the nucleation and growth aspects. In the process of lithium nucleation, the effects of overpotential and current exchange density in different electrolytes were investigated. A higher exchange current density prolongs an EDL’s charging time, resulting in more electrolyte decomposition to form the initial SEI. In addition, a higher overpotential produces a finer and denser deposition morphology. In lithium nuclei growth, the growth direction of individual lithium particles is perpendicular to the Cu substrate, such that an elongated lithium deposition morphology could be observed in the previous work. This phenomenon is attributed to the difference between the top and side of the SEI of the lithium-particle during deposition, and this difference continues till the dissolution of the deposited lithium, causing a doughnut-like dissolution morphology. Furthermore, three growth modes of lithium particles were proposed, marked as preferential growth, merged growth, and independent growth. A lithium deposition schematic was established to describe the process clearly. This study is meaningful for understanding the principle of lithium deposition, providing a theoretical basis for solving the problem of lithium dendrite growth.

## Figures and Tables

**Table 1:** Composition of electrolytes.

Electrolytes	Marks
1 M LiTFSI DOL:DME (1:1)	LS001
1 M LiPF_6_ EC:DMC (1:1)	LB001
1 M LiTFSI DOL:DME (1:1) + 2% wt. LiNO_3_	LS009
